# Image-based siRNA screen to identify kinases regulating Weibel-Palade body size control using electroporation

**DOI:** 10.1038/sdata.2017.22

**Published:** 2017-03-01

**Authors:** Robin Ketteler, Jamie Freeman, Francesco Ferraro, Nicole Bata, Dan F. Cutler, Janos Kriston-Vizi

**Affiliations:** 1 MRC Laboratory for Molecular Cell Biology, University College London, Gower Street, London WC1E 6BT, UK

**Keywords:** Fluorescence imaging, High-throughput screening, Kinases, Inhibitory RNA techniques

## Abstract

High-content screening of kinase inhibitors is important in order to identify biogenesis and function mechanisms of subcellular organelles. Here, we present a human kinome siRNA high-content screen on primary human umbilical vein endothelial cells, that were transfected by electroporation. The data descriptor contains a confocal fluorescence, microscopic image dataset. We also describe an open source, automated image analysis workflow that can be reused to perform high-content analysis of other organelles. This dataset is suitable for analysis of morphological parameters that are linked to human umbilical vein endothelial cell (HUVEC) biology.

## Background & Summary

Microscopy-based high-content screening has emerged as a powerful method to interrogate biological pathways and to identify novel genes involved in cellular processes^1^. A common method that has found wide spread application is using short interfering RNA libraries^2,3^, and more recently, the use of CRISPR-based arrayed libraries has been suggested^4^. One caveat in the use of such methods is the high degree of variability between samples, often due to inherent biological variation or technical limitations. Several computational methods have aimed to reduce such variability, which can be applied post-screening to extracted datasets and improve analysis without the need to re-acquire images^5,6^. Of interest, many high-content datasets only report few parameters and defeat the concept of multiparametric analysis that is possible with acquired images^7^. Thus, it can be useful to publish images from high-throughput screens in order to enable re-analysis and generation of novel insights by *de novo* analysis using alternative parameters. Furthermore, the analysis of a large number of objects in an image dataset sometimes enables unexpected discoveries. For instance, we have previously assessed the size of Weibel-Palade bodies (WPB) and identified a periodic length distribution when a large number of objects were analysed^8^. Weibel-Palade bodies are secretory storage granules in endothelial cells that have an important role in primary haemostasis. WPBs are the endothelial storage organelles of von-Willebrand factor (vWF), a large adhesive glycoprotein that is released upon vascular injury and promotes the recruitment of platelets to the site of injury, thereby initiating primary haemostasis. WPBs can be visualised in cells by vWF immuno-staining and therefore factors that regulate this WPB formation can be identified by high-content screening approaches. We have previously monitored the length of WPBs in cells by high-throughput morphometry and identified a role for the Golgi ribbon in determining the size of these structures. This has led to novel insights into the biogenesis and function of these organelles with the concept of vWF cargo ‘quanta’ that form at the Golgi and can be packaged together during biogenesis WPBs, generating organelles of varying sizes^8^. Following this work, we have explored the possibility that WPB size may be regulated by kinases and performed a kinome siRNA screen in primary Human Umbilical Vein Endothelial Cells (HUVECs). Other works have identified GRK2 (ref. 9) and PI4-kinase^10^ kinases as regulators of WPB biogenesis and vWF release. We used electroporation as a technique to improve transfection efficiency into primary cells^11^ and applied an image analysis pipeline that is suitable for the analysis of intracellular organelle size and number.

**Figure 1:**
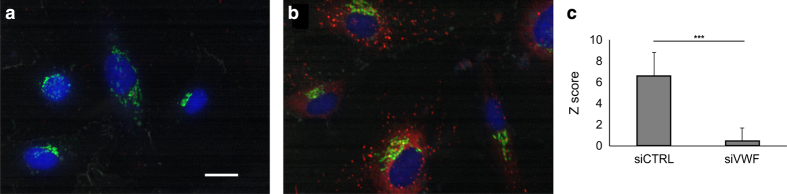
Representative WPB phenotypes and quantification of positive and negative controls. (**a**) siRNA (negative control) (**b**) siRNA knockdown of vWF (positive control). Nuclei (blue), *trans*-Golgi network (green), WPB (red) and plasma membrane (grey) were stained in HUVEC cells. Scale bar 20 μm. The figures show representative, cropped regions of fields of view, that allowed the proper visualization of the small WPBs. (**c**) The intensity Z scores (3_TotalWPBrawIntDenPerCellNR) of negative control wells (siCTRL) are significantly higher than that of positive control wells (siVWF). Mean±S.D. plotted for 88 independent biological replicates for each condition, two-tailed Student’s two sample t-test *P*<2.2e-16.

We recorded a dataset of 10,560 images in four microscope channels monitoring WPBs, nuclei, *trans*-Golgi network and plasma membrane. We developed an image analysis pipeline to determine the size and length of Weibel-Palade Bodies. Positive (siRNA knockdown of vWF, Fig. 1a) and negative (control siRNA, Fig. 1b) controls were used to determine the suitability of this assay for monitoring changes in a large-scale screening format. Overall, more than 10 million objects were identified as WPBs and measured for intensity, Feret’s diameter, and other parameters, listed in Table 1. In addition, we also monitored key cellular features such as cell number to account for effects on cell viability, a common phenotype observed in siRNA knockdown screens. We confirmed GRK2 as a positive hit and identified other potential candidate kinases that regulate WPB length and number (Table 2). When assessing the reproducibility among biological replicates, we observed a high level of variability between replicates that might reflect technical limitations of the electroporation. Another possibility is that the low dynamic range observed in this assay reflects inherent features of the underlying biology, e.g. that multiple kinases may only have a minor impact on WPB formation. Nonetheless, this dataset can be used to extract other morphological features that are not directly linked to WPB biology. Alternatively, post-screening methods that enable the separation of on- and off-target effects may greatly benefit the analysis. We therefore make this dataset publicly available to encourage re-analysis by means of alternative methods.

In summary, we present here data from a siRNA screen targeting the human kinome in order to identify regulators of Weibel-Palade body formation. The screen was performed in primary human umbilical vein endothelial cells (HUVECs), a cell type that has been difficult to transfect with common lipofection methods^12^. We therefore used electroporation with the 96-well nucleofector machine and achieved good knockdown efficiency using the positive control (vWF-targeting siRNA) (Fig. 1). We developed an image analysis pipeline in ImageJ and identified several kinases that regulate the area, number, length and intensity of WPB objects. We therefore propose that the formation of WPBs is regulated by kinases. Further, we demonstrate that electroporation can be used in a high-content screening workflow to generate data from difficult to transfect cell types. And finally, we present an image analysis workflow for the identification and analysis of key features of cellular organelles, in this case WPBs, that can be applied to other sub-cellular structures in high- or low-throughput settings.

## Methods

### Electroporation and Screening

Primary HUVECs were transfected in suspension using the Lonza/Amaxa nucleofector 96-well shuttle. The human kinome MISSION siRNA kinase panel library from Sigma-Aldrich was used in this study (Supplementary Table 1). Three oligonucleotide pairs were provided for each gene in a total of 33 plates. A pool of three siRNA molecules targeting the same gene was generated by mixing equal amounts of the three individual siRNA oligonucleotides together, generating 11 ‘pool’ plates that were used for screening. Control siRNAs were custom synthesised, as described^8^. An siRNA targeting firefly luciferase was used as a negative control and siRNA targeting vWF as positive control. Cells were thawed and cultured for one week prior to the screen, low passage, P3-P5 cells were used. Briefly, 75,000 cells were mixed with 20 μl nucleofection buffer and the pre-defined HUVEC nucleofection protocol on the instrument was used for electroporation of 30 pmol siRNA, in agreement with standard nucleofection protocols used in other screens^12^. Cells were seeded into 96 well, optically clear bottom, tissue culture treated, sterile, polystyrene (Nunc, product number 167008) microplates that were pre-coated with gelatin and allowed to adhere overnight. Media was replaced the following day, and cells were fixed in 4% PFA and stained 48 h after electroporation. The 48 h time frame is well in line with recommendations for siRNA screening (e.g. Martin *et al.* ^13^, which recommends 48–120 h for screening). Immunostaining was performed as described^8^. Briefly, we used Hoechst33342 for nuclear staining, rabbit anti-vWF antibody for WPBs, sheep anti-TGN-46 antibody for the trans-Golgi network, and mouse anti-ve-Cadherin antibody for plasma membrane staining. The following antibodies were used: anti-vWF, rabbit polyclonal (Dako, cat. no. A0082), anti-TGN46, sheep polyclonal antibody (AbD Serotec, cat. no. AHP500) and anti-VE-cadherin, mouse monoclonal antibody (BD Biosciences, cat. no. 555661).

### High-content image acquisition

Images were acquired with a spinning disk confocal Opera LX plate reader microscope (PerkinElmer) with Olympus 40x LUCPLFLN (NA=0.6) air lens at four fluorescence channels using 365 nm, 488 nm, 561 nm and 640 nm excitation wavelengths respectively. Camera binning was not used, resulting in a nominal pixel size of 0.1615 μm. We worked with confluent cells, 70–100 cells were imaged in the 5 fields of view of the wells.

The manufacturer’s recommended procedures (described in Opera Quick Guide 1.8.1, product number HH10940200) and Opera Adjustment Plate (PerkinElmer, product number HH10000650) was used for optical corrections. In order to compensate the systematic uneven brightness distribution of the optics, reference images were taken for flatfield correction. The ‘fish eye effect’ and offset between channels was compensated using bead images for skewcrop analysis. Both flatfield correction and skewcrop analysis was done before image acquisition of each experiment. Five field of views (FoVs) were acquired in each well.

The microscopy exposure times and laser powers were used as follows. 250 ms exposure time was used at the 365 nm channel for nuclear imaging, 2000 ms with 3620 μW laser power at the 488 nm channel for *trans*-Golgi network (TGN46), 2000 ms with 1640 μW laser power at the 561 nm channel for WPB (vWF staining) and 2000 ms with 862 μW laser power at the 640 nm channel for plasma membrane (VE-cadherin). The screen was done in two batches (pools A-F on one day and G-K on another day).

The 4-channel, fluorescence, 12 bit depth images were acquired as Opera *.flex files and analysed after conversion into *.tif format by the Acapella FlexToVolocity.script.

### High-content analysis

#### Image processing.

*Hardware specifications of the high-content-analysis computer*: A high-performance computer was built on a Z8PE-D18 server motherboard (Asus) equipped with 145 GB memory, two Xeon E5520 CPUs (Intel) running at 2.27 GHz clock rate.

#### ImageJ macro

**Figure 2:**
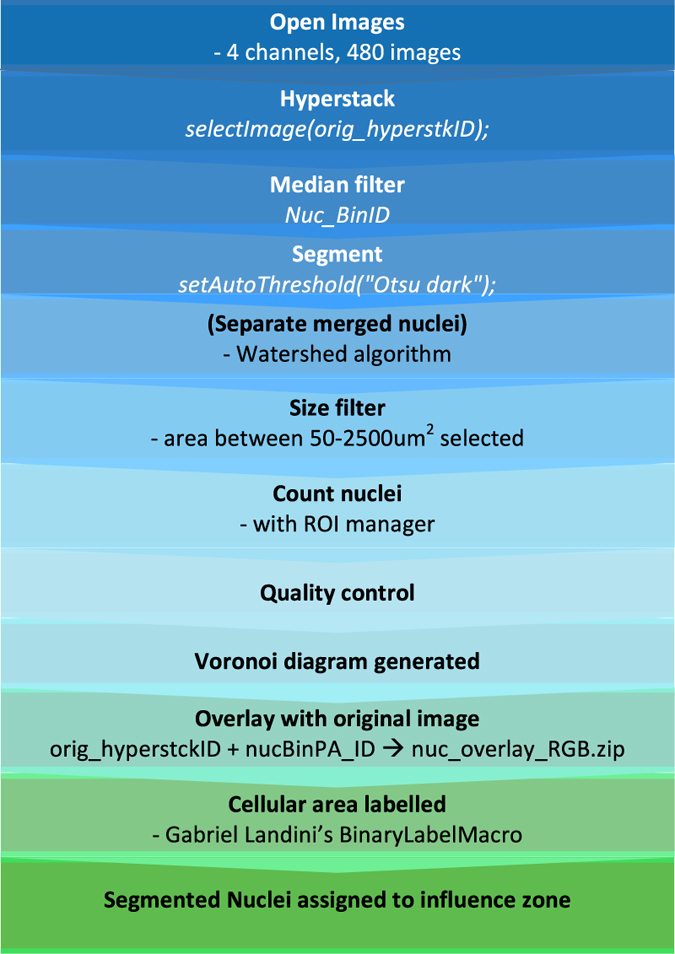
Diagram depicting the image processing workflow by ImageJ.

The macro (Fig. 2) uses 45 GB memory, for automatically analysing the 480 images of a whole 96 well plate, in 30 min. For the Bernsen thresholding, it requires Gabriel Landini’s Auto Local Threshold plugin (http://imagej.net/Auto_Local_Threshold) to be installed to ImageJ. Alternatively, the Auto Local Threshold plugin is integrated into Fiji (http://fiji.sc). The macro requires subfolders in the results folder named as: influzone, overlay_nuc_RGB, overlay_vWF_RGB, Results_Features, Results_Nuc_Features, Results_WPB_cell_ID and RoiSet.

The macro has two main parts. The first part segments the nuclear channel and approximates individual cellular area using the midlines between nuclei. The second part analyses the WPB channel.

The nuclear channel was pre-processed using a Median filter with 2 pixel radius in order to reduce the inherent noise in the image and smoothen the nuclear contours. The latter is a necessary step because of the separation mechanism of touching nuclei. Watershed algorithm was used to separate merged nuclei based on convexity and smoothened contours eliminate separating artefacts caused by minor concavities on a coarse nuclear contour. A size filter eliminates artefact objects that cannot be considered as nuclei, being too small (<50 μm^2^) or too large (<2500 μm^2^).

Quality control (QC) plays an important role in automated image processing. Contour overlays on the original images serve as a convenient, quick visual QC method and helps to find the optimal parameters at the image processing step.

As a QC of the nuclear segmentation, the contours of the nuclei overlaid on the original nuclear images were automatically saved by the macro.

Delaunay Triangulation was used to generate a Voronoi diagram i.e. the midlines between nuclei centroids in order to generate influence zones of nuclei that approximate individual cell contours. There are advantages of this approach: it is simple, allows single cell analysis, requires nuclear stain only, and is computationally cheap. It is limited though: it follows the real cell boundaries only if those lay on the midline between the nuclei and it assumes that the cells form a confluent monolayer. Practically, the latter shortcoming is significant only if the precise quantification of cellular area is needed. In this screen this feature was only used as a proof of concept to provide a simple initial approach for the development of a more precise plasma membrane delineating pipeline. The Voronoi mesh was overlaid on the original nuclear channel for each image and the result was saved as an image stack (see the *_nuc overlay_RGB.zip files (Data Citation 1)).

In order to assign its cell identifier to each WPB pixel, the cellular area was labelled using code from Gabriel Landini’s BinaryLabelMacro (http://sites.imagej.net/Landini/plugins/Morphology/BinaryLabelMacro.ijm-20140627120652). Our image processing workflow uses grey shades to label cells. An 8 bit image allows the use of 2^8^=256 grey shades, that can be a rate limiting factor when the image has more than 256 cells. Therefore, we use 16 bit images, that allow the labeling of more than 256, maximum 2^16^=65535 cells in an image. The labels were encoded into 16 bit range values, with 0 for the separating Voronoi mesh. The 16 bit depth labelling was used for computational purposes, automated contrast stretch was applied when the labelled images were visualised and saved in RGB stack format (see the *_influzone.zip files (Data Citation 1)).

Furthermore, the segmented nuclei were assigned to their corresponding influence zone by measuring the mean intensity on influence zone image. The results were saved in the *_Results_Nuc_Features.csv files (Data Citation 1).

**Figure 3:**
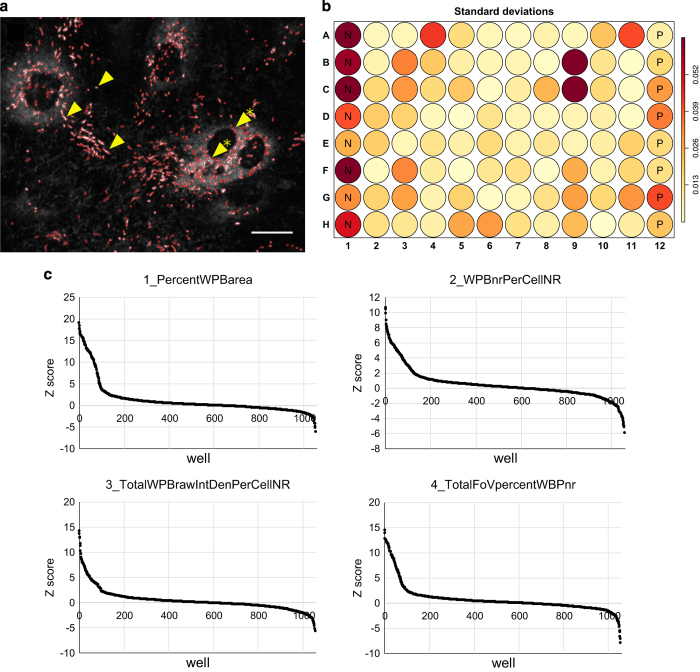
Electroporation-based siRNA screen result. (**a**) Segmentation quality control overlay image. The red contour indicates the boundary of segmented WPBs. Representative image, the overlay of all processed images are available in the repository as *_vWF_overlay_RGB.zip files and the WPB contours as *_RoiSet.zip files (Data Citation 1). Arrows denote correctly segmented representative WPBs, arrows with star denote non-WPB vWF objects as segmentation artefacts. Scale bar 20 μm. (**b**) Reproducibility is quantified by calculating the standard deviation of per well values across the replicates. A representative plate heatmap is presented here and the data for all plates are available in the repository as components of the HTML reports. Negative control wells are labelled with ‘N’ and ‘P’ stands for positive control wells. (**c**) Distribution of the 1056 well’s Z scores in the 1_PercentWPBarea dataset. All Z score values are available in the HTML reports (Data Citation 1).

At this point, the image processing workflow was ready for the WPB channel quantification. A copy was created about the second (WPB) channel in order to preserve the original pixel intensities so intensity features can be measures after the segmentation. Subsequently, the thresholding was performed on the duplicated stack. Noise reduction was performed using the rolling ball algorithm^14^ with 1 pixel as rolling ball radius parameter and using sliding paraboloid correction. We used a Bernsen local thresholding^15^ with the default contrast threshold parameter value (15) on the 8 bit converted WPB images because we found that it provided us with an acceptable precision. The contrast threshold is the only parameter value that might need adjustment in the entire image processing macro. If the fluorescence signal is too weak, then the Bernsen algorithm becomes ‘too sensitive’ and detects even the out of focus and background pixels. In that case the user needs to set the algorithm ‘insensitive’ by increasing the contrast threshold parameter from the default 15 value to 30 or 50. The WPB pixels overlapping the Voronoi mesh were deleted in order to avoid the ambiguous situation, when a WPB crosses the approximated cell boundary. The segmented WPBs were saved as ImageJ region of interest (ROI) sets for an entire plate of image stack as *_RoiSet.zip files (Data Citation 1) (Fig. 3a).

During feature extraction, both morphology and intensity-based WPB features were quantified (Table 1). Objects larger than 10 μm^2^ were considered as virtually merged WPB clumps, that the microscope was unable to resolve separately. Therefore, those large objects were omitted using a size filter. The measured features with the well and FoV identifier of each WPB were saved in the comma separated *_Results_Features.csv ASCII files (Data Citation 1).

The WPB labelling was an essential step in order to assign each organelle to its influence zone. The mean pixel intensity of each WPB object was measured on the influence zone image. The number of rows (WPBs) in the resulted table was equal to the corresponding Results Feature files. The result of the measurements was saved in comma separated ASCII files named as *_Results_WBP_cell_ID.csv (Data Citation 1).

For QC purpose, the contours of the segmented and size filtered WPBs were overlaid in red on their original greyscale images and was saved as stack in RGB format in the *_vWF_overlay_RGB.zip (Data Citation 1) files. The overlay image facilitated visual inspection in order to identify vWF-rich areas, that were segmented as non-WPB artefacts (Fig. 3a).

### R script

**Figure 4:**
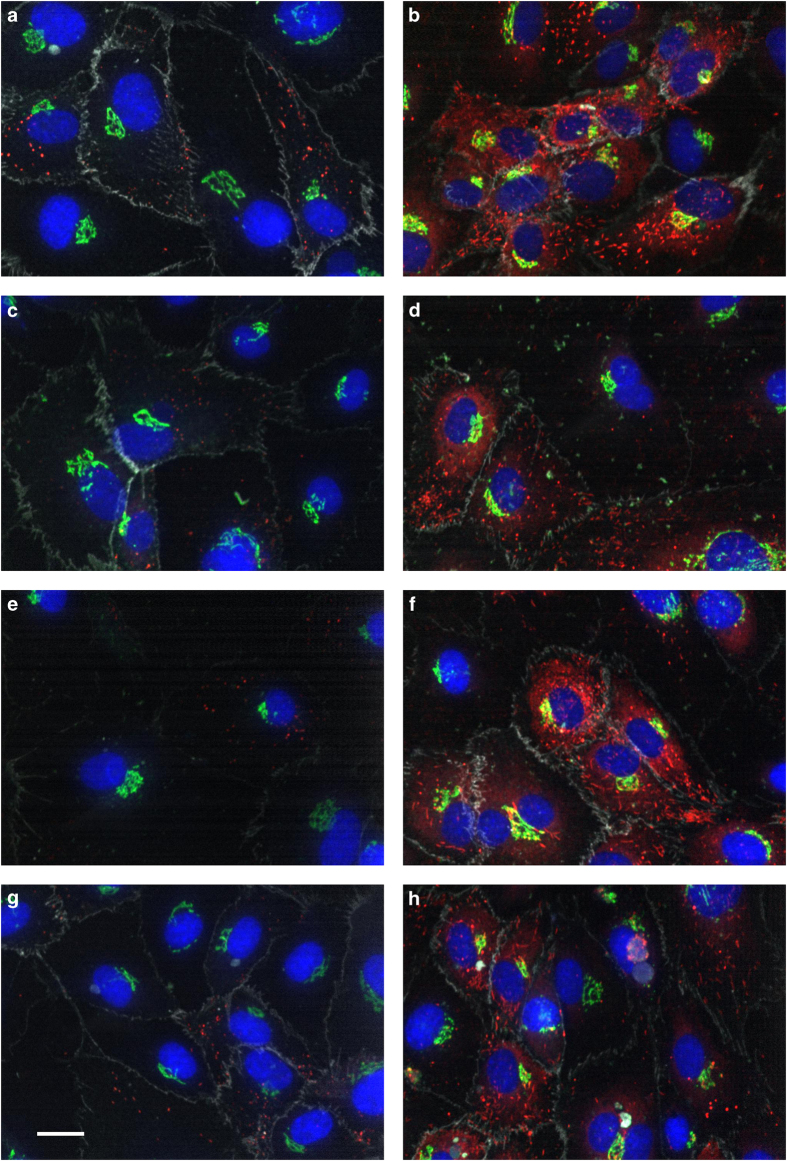
Representative control and hit phenotypes for all four biologically relevant parameters. siCTRL (left panels) and top hit phenotypes (right panels) for WPB area ratio control (**a**), top hit kinase DMPK (**b**), WPB number control (**c**), top hit kinase TRRAP (**d**), WPB intensity control (**e**), top hit kinase MAPK12 (**f**) and ratio of WPB with Feret diameter longer than 1.5 μm control (**g**), top hit kinase MAP4K2 (**h**), respectively. Scalebar 20 μm.

The morphological and intensity results of the WPBs were stored in ASCII tables and were evaluated statistically with Rscript.R (Data Citation 1) using R^16^, the well-established, open source statistical software (http://r-project.org). The R scripts calculated single values per well in each plate for:

1. area ratio of WPBs with Feret’s diameter larger than 1.5 μm (1_PercentWPBarea),
2. WPB number per cell (2_TotalWPBnrPerCellNR),
3. WPB intensity per cell (3_TotalWPBrawIntDenPerCellNR, Fig. 1c) and
4. ratio of WPB number with Feret’s diameter larger than 1.5 μm (4_TotalFoVpercentWPBnr).

Representative phenotypes for hits versus controls are shown in Fig. 4.

In order to calculate the 1_PercentWPBarea feature for each well, first the total WPB was calculated (sum(FoVfeature$Area)). Then the WPBs were selected with Feret’s diameter larger than 1.5 μm (over15umfeature), followed by calculating the total area of those WPBs (sum(over15umfeature$Area)) and their ratio (FoVpercentWBParea[f]). The mean of the 5 FoVs resulted the single PercentWPBarea value in each well.

The 2_TotalWPBnrPerCellNR feature was also calculated as a mean of five FoV ratios in each well. In each FoV the total number of WPBs (FoVTotalWPBnr) was devided by the total number of cells (FoVcellNR). The number of cellID values was extracted from the *_Results_WBP_cell_ID.csv file (Data Citation 1).

The WPB intensity per cell, 3_TotalWPBrawIntDenPerCellNR, was a ratio of the total intensity of all WPBs (sum(FoVfeature$RawIntDen)) and the total number of cells (FoVcellNR). The mean of the five FoVs gave the single value per well.

The 4_TotalFoVpercentWPBnr was calculated as a ratio of the WPB number with Feret’s diameter larger than 1.5 μm (length(over15umfeature$Area)) and the total number of WPBs (FoVTotalWPBnr) in a given FoV. The per-well value was calculated as the mean of the 5 FoV results.

The results of each plate were saved separately as ASCII files into subfolders named as 1_PercentWPBarea, 2_TotalWPBnrPerCellNR, 3_TotalWPBrawIntDenPerCellNR and 4_TotalFoVpercentWPBnr respectively.

The files have a 3 column data structure, that is required by the cellHTS2 package. The plate identifier in the first column (arbitrary string), the well identifier in the second column (e.g. A01, H12) and the measured/calculated single value in the third column.

### Bioconductor cellHTS2 script

We used the cellHTS2 (ref. 17) package from Bioconductor in custom R scripts for statistical analysis. The workflow was prepared following the sample workflow described in ‘End-to-end analysis of cell based screens: from raw intensity readings to the annotated hit list’ (last retrieved on 09/02/2017 from https://www.bioconductor.org/packages/3.3/bioc/vignettes/cellHTS2/inst/doc/cellhts2Complete.pdf).

The file cellHTS2_VWF_1_PercentWPBarea.R contains an explanation of the method used for analysis. Initially, the working folder and the path to dataset was specified (these two folders can be identical). The dataset path was the folder where the Rscript.R result files were saved. The Platelist.txt, Plateconf.txt and Description.txt files (Data Citation 1) were prepared based on the sample workflow instruction. The dataset was normalised by the B score^18^ method. Following normalisation, the Z score (Fig. 3c) of each well was calculated and a report was generated in HTML format.

### Code availability

The image analysis was performed under a 64 bit version of Kubuntu Linux 12.04, ImageJ version 1.50 f and Fiji^19^, R version 3.2.5, cellHTS2 version 2.30.0.

The ImageJ_macro_vWF_HCS.ijm file is available at Harvard Dataverse repository (Data Citation 1).

The Rscript.R file is available at Harvard Dataverse repository.

All HTS statistical analysis R source code files (e.g. cellHTS2_VWF_1_PercentWPBarea.R) are available at Harvard Dataverse repository.

## Data Records

The data associated with this work is available at the Harvard Dataverse repository (Data Citation 1).

All .zip files were renamed ‘.zip_’ to circumvent the automatic extracting function of Harvard Dataverse repository. When downloading this data, the researcher needs to rename all files to *.zip again.

Image data files are named as A1.zip-K2.zip. After downloading, these files need to be unzipped into different folders.

The ImageJ_macro_vWF_HCS.ijm source code ASCII file contains the ImageJ/Fiji macro that perform the automated image analysis of a plate’s image set.

Data resulting from the Delaunay Triangulation can be found in the *_influzone.zip and in *_nuc-overlay_RGB.zip files. The *_vWF_overlay_RGB.zip files contain the segmentation contours of the WPBs overlaid on the original images and can be used for visual inspection QC. These contain images of a plate as stacks, do not require unzipping and can be opened directly with ImageJ or Fiji by the File/Open menu.

*_Results_Nuc_Features.csv comma separated value ASCII files: the filename contains the plate number, the field ‘Label’ contains the well (row and column number) and FoV number, the field ‘Mean’ contains the influence zone identifier number of a given nucleus.

Segmented WPB contour data can be found for each plate in *_RoiSet.zip files. These files can be opened in ImageJ or Fiji using the Open function in ROI Manager.

The *_Results_Features.csv comma separated value ASCII files contain the measured WPB features.

The WPB labels, that connect them to a specific cell are stored in the *_Results_WBP_cell_ID.csv comma separated value ASCII files.

The Rscript.R source code ASCII file contains the statistical analysis of the measured WPB featured. It is a script written in R language.

The cellHTS2_VWF_1_PercentWPBarea.R, cellHTS2_VWF_2_TotalWPBnrPerCellNR.R, cellHTS2_VWF_3_TotalWPBrawIntDenPerCellNR.R and cellHTS2_VWF_4_TotalFoVpercentWBPnr.R ASCII files are R scripts. Those contain the source code of the HTS statistical analysis.

The 1_PercentWPBarea_cellHTS2_SciData.zip,

2_TotalWPBnrPerCellNR_cellHTS2_SciData.zip,

3_TotalWPBrawIntDenPerCellNR_cellHTS2_SciData.zip and

4_TotalFoVpercentWBPnr_cellHTS2_SciData.zip files contain the input data, HTS analyses and HTML format reports, generated by their respective HTS statistical analysis R scripts. After downloading, these files need to be unzipped into different folders. The subfolder called as report-VWF* contains an index.html file that can be opened in any web browser.

## Technical Validation

WPB segmentation: The *_vWF_overlay_RGB.zip files contain the segmentation contours of the WPBs overlaid on the original images and can be used for visual inspection QC.

In order to assess the quality of the screen Z’ factors were calculated to both replicates of all 4 WPB HTS features, and available in the respective HTML reports.

Standard deviation was calculated across the replicates for all of the four WPB HTS features. It can be found in the HTML reports as reproducibility plate heatmaps (Fig. 3b).

## Usage Notes

The user must rename the *.zip_ files to *.zip. The extension renaming was necessary in order to circumvent the automated decompress feature of Harvard Dataverse repository.

## Additional Information

**How to cite this article**: Ketteler, R. *et al.* Image-based siRNA screen to identify kinases regulating Weibel-Palade body size control using electroporation. *Sci. Data* 4:170022 doi: 10.1038/sdata.2017.22 (2017).

**Publisher’s note**: Springer Nature remains neutral with regard to jurisdictional claims in published maps and institutional affiliations.

## Supplementary Material



Supplementary Table 1

## Figures and Tables

**Table 1:** WPB features quantified by ImageJ

**label**	**description**
Area	Area of a WPB object in μm^2^.
Mean	Average grey intensity of a WPB object [relative fluorescence unit r.f.u.]
StdDev	Standard deviation of a WPB object pixel intensity [r.f.u.].
Min	Minimum intensity of a WPB object [r.f.u.].
Max	Maximum intensity of a WPB object [r.f.u.].
X	The X coordinate of a WPB object’s centroid [pixel].
Y	The Y coordinate of a WPB object’s centroid [pixel].
Perim.	Perimeter of a WPB object [μm^2^].
Major	Length of primary axis of the best fitting ellipse around a WPB object [μm].
Minor	Length of secondary axis of the best fitting ellipse around a WPB object [μm].
Angle	Angle between the horizontal axis of the image and the primary axis of the best fitting ellipse around a WPB object [degree].
Circ.	Circularity shape descriptor of a WPB. Circ.=1 means a perfect circle.
Feret	The longest diameter between any two points along a WPB’s boundary [μm].
IntDen	The product of a WPB’s area and mean grey intensity [r.f.u.].
Median	Median grey intensity of a WPB object [r.f.u.].
RawIntDen	The sum of the values of the pixels in a WPB [r.f.u.].
Slice	The number of image in an image stack.
FeretX	The X coordinate of the end of a WPB’s Feret diameter [pixel].
FeretY	The Y coordinate of the end of a WPB’s Feret diameter [pixel].
FeretAngle	Angle between the horizontal axis of the image and the Feret’s diameter of a WPB object [degree].
MinFeret	The shortest diameter between any two points along a WPB’s boundary [μm].
AR	Aspect ratio is a shape descriptor of a WPB, means the ratio of the length of the primary and secondary axes of a WPB’s fitted ellipse.
Round	Roundness is a shape descriptor of a WPB, the inverse of AR.
Solidity	The ratio between a WPB’s area and convex area.
The features listed as headings and are described at the ImageJ documentation website: https://imagej.nih.gov/ij/docs/guide/146-30.html#toc-Subsection-30.7 (accessed on 09/02/2017).	

**Table 2:** GRK2 is identified as a hit regulating WPB number

**Z score**	**plate**	**well**	**GeneSymbol**
6.88	6	A07	DYRK4
5.33	11	E05	LMTK3
5.01	6	B05	CDK10
3.52	11	E02	SBK1
3.47	11	C04	PRKG1
3.31	6	F02	PIP5K1B
3.16	6	C05	CDK10
3.12	11	F03	PIP5K1A
3.03	6	B02	BRD3
2.95	11	B04	RAPGEF3
2.92	11	E07	CSNK2A1
2.85	6	F11	MAP4K4
2.85	6	B08	HERC2
2.76	8	A08	TRIM33
2.69	7	F04	HIPK3
2.64	8	A10	TRPM7
**2.57**	**1**	**A03**	**ADRBK1**
2.57	6	F03	MAP4K3
2.56	8	C09	PANK1
2.51	9	F04	ADCK1
2.5	8	A02	SGK3
2.47	6	C06	CDC2L5
2.34	11	F06	DYRK1B
2.28	6	G05	CASK
2.24	3	B09	NEK2
2.24	3	F11	PCTK2
2.23	6	G07	STK19
2.22	1	A04	AMHR2
2.21	10	C06	NEK7
2.17	8	B05	TBK1
2.16	9	E07	WNK2
2.16	10	C09	PRPS1L1
2.16	9	E08	SGK269
2.16	6	G08	CDKL2
2.08	2	A06	MARK2
2.06	11	D04	PRKDC
2.04	2	B05	DMPK
2.02	7	H05	RAPGEF3
2.02	6	A06	STK16
2.01	10	B04	EVI5L
Hitlist show positive hits, that were thresholded as Z score>2. GRK2 (syn. ADRBK1) was confirmed as one of the top hits.			
