# Corrigendum: Image-based siRNA screen to identify kinases regulating Weibel-Palade body size control using electroporation

**DOI:** 10.1038/sdata.2017.86

**Published:** 2017-06-27

**Authors:** Robin Ketteler, Jamie Freeman, Nicola Stevenson, Francesco Ferraro, Nicole Bata, Dan F Cutler, Janos Kriston-Vizi

*Scientific Data* 4:170022 doi:http://dx.doi.org/10.1038/sdata.2017.22 (2017); Published 1 Mar 2017; Updated 27 Jun 2017

The authors regret that Nicola Stevenson was omitted in error from the author list of the original version of this Data Descriptor. This omission has now been corrected in the HTML and PDF versions of this Data Descriptor, as well as the accompanying Corrigendum.

Contributions

R.K., D.C., F.F. and J.K.V. designed and conceived the study. J.F., N.S. and F.F. performed experiments. J.K.V. performed all statistical and image analysis. R.K., J.K.V. and N.B. wrote the manuscript.

